# Higher biomass accumulation by increasing phosphoribosylpyrophosphate synthetase activity in *Arabidopsis thaliana* and *Nicotiana tabacum*

**DOI:** 10.1111/j.1467-7652.2007.00314.x

**Published:** 2008-04

**Authors:** Silke Koslowsky, Heike Riegler, Eveline Bergmüller, Rita Zrenner

**Affiliations:** Max-Planck-Institut für Molekulare Pflanzenphysiologie, Am Mühlenberg 114476 Potsdam Golm, Germany

**Keywords:** metabolic regulation, phosphoribosylpyrophosphate synthetase, plant growth, transgenic plant

## Abstract

Plants are able to produce all the organic compounds required for development and growth. As developmental processes and metabolic pathways use a common resource pool, the tight regulation of the distribution of metabolites between growth, production of defence compounds and storage products can be assumed. A transgenic approach was used to investigate the importance of supplying the key intermediate phosphoribosylpyrophosphate (PRPP) for plant growth and biomass accumulation in the model plant *Arabidopsis thaliana* and in *Nicotiana tabacum*. For this purpose, the *Ashbya gossypii* genes coding for either PRPP synthetase (*PRS*) or a mutated variant of the same gene were over-expressed under the control of a constitutive promoter. It was shown that increased PRS activity in *A. thaliana* or *N. tabacum* leads to a substantial increase in biomass accumulation under different standardized growth conditions. Growth enhancement was accompanied by significant changes in the amount of sugars and other metabolites. This study provides evidence that the supply of PRPP co-limits growth rates, and has obvious implications for biotechnological strategies aiming to increase plant biomass as an alternative renewable energy source.

## Introduction

Plants are photoautotrophic organisms that can produce all the organic compounds needed for development and growth. Over recent years, many factors that influence the cell and organ growth of plants have been identified, and the molecular functions of growth-related proteins have started to be elucidated (Anastasiou and Lenhard, 2007). As developmental processes and metabolic pathways use a common resource pool, and both processes respond to changes in environmental energy and resource supplies, it is evident that resource availability may have a direct influence on cell proliferation and growth. This close interrelation has recently been demonstrated by Baldet *et al*. (2006), who showed that fruit load reduction in tomato plants resulted in increased photoassimilate availability and increased growth rates in all other plant organs, including roots, stems, leaves, flowers and other fruits. Therefore, a tight regulation of the distribution of metabolites between growth, production of defence compounds and storage products can be assumed. Faster growing *Arabidopsis* accessions are characterized by lower levels of central metabolites, such as carbohydrates and amino acids, showing that faster growth draws on and depletes the levels of these central resources (Cross *et al*., 2006). A detailed knowledge of the relations between metabolism and biomass accumulation can be expected to yield powerful novel tools to accelerate plant breeding programmes in the future. Furthermore, metabolic profiling in the model *Arabidopsis* using a recombinant inbred line (RIL) population has recently revealed a close link between biomass and a specific combination of metabolites (Meyer *et al*., 2007). This demonstrates the predictive power of metabolite composition as a biomarker for biomass production, opening up new opportunities for plant breeding.

Phosphoribosylpyrophosphate (PRPP) is a key intermediate in metabolism, essential for nucleotide production (Zrenner *et al*., 2006) and the synthesis of the pyridine nucleotide cofactors NAD and NADP (Noctor *et al*., 2006) and the amino acids histidine (Stepansky and Leustek, 2006) and tryptophan (Radwanski and Last, 1995). PRPP is needed in the respective *de novo* synthesis pathways, but is of equal importance in the salvaging processes of nucleobases and pyridines. Thus, the sufficient availability of PRPP is a crucial prerequisite for any metabolic process and, subsequently, for growth. Phosphoribosylpyrophosphate synthetase (ATP:d-ribose-5-phosphate pyrophosphotransferase; EC 2.7.6.1; PRS) catalyses the synthesis of PRPP from ribose 5-phosphate (R5P) and ATP. PRSs are a family of enzymes grouped into three classes on the basis of their dependence on phosphate ions for activity, their allosteric regulation and their specificity for diphosphoryl donors. Class I proteins are found in all organisms and require Mg^2+^ and phosphate for activity, can be inhibited allosterically by ADP and prefer ATP or dATP as diphosphoryl group donor (Krath and Hove-Jensen, 2001a). Class II PRSs are found exclusively in plants, do not depend on phosphate for activity, lack the allosteric regulation of ADP and have a much broader specificity for the diphosphoryl donor (Krath and Hove-Jensen, 1999, 2001b). Recently, a novel class III PRS has been identified from *Methanocaldococcus jannaschii* resembling class I properties, but also lacking an allosteric site for ADP (Kadziola *et al*., 2005).

In previous experiments, it has been shown that, with decreased nucleotide *de novo* synthesis, the growth of potato and tobacco plants is reduced without further pleiotropic effects (Schröder *et al*., 2005). In this study, our aims were to globally increase nucleotide availability by increasing the supply of the common precursor PRPP, and to investigate its influence on plant growth and biomass accumulation. For this purpose, a transgenic approach was used to increase the PRPP supply in the model plant *Arabidopsis thaliana* and in *Nicotiana tabacum*, representing a plant that achieves a larger vegetative biomass. An *Ashbya gossypii* gene coding for PRS (*PRS*) and a mutated variant of the same gene were expressed in transgenic plants using constitutive expression from the 35S promoter of the cauliflower mosaic virus. It was shown that increased PRS activity leads to a substantial increase in plant growth and biomass accumulation under different standardized growth conditions. This provides evidence that PRPP supply co-limits growth rates, and has obvious implications for biotechnological strategies aiming to increase plant biomass as an alternative energy source.

## Results and discussion

### Production and selection of transgenic plants with expression of a *PRS* gene from *As. gossypii*

**Figure 1:**
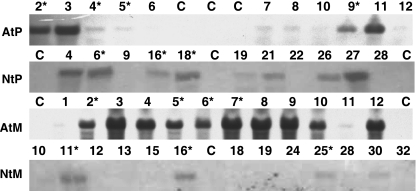
Expression of the *Ashbya gossypii* phosphoribosylpyrophosphate synthetase (*PRS*) gene in leaves of *Arabidopsis thaliana* and *Nicotiana tabacum* transformants. Plants were grown on selection medium, and resistant plantlets were transferred to soil and grown under standard cultivation conditions. The leaves of these plants were analysed 3–5 weeks after transfer for steady-state *PRS* mRNA levels using the full-length cDNA coding for PRS as hybridization probe. The same amounts of RNA were analysed. AtP, *A. thaliana* transformed with a construct to express the wild-type *PRS* gene of *As. gossypii*; NtP, *N. tabacum* transformed with a construct to express the wild-type *PRS* gene of *As. gossypii*; AtM, *A. thaliana* transformed with a construct to express a mutant form of the *PRS* gene of *As. gossypii*; NtM, *N. tabacum* transformed with a construct to express a mutant form of the *PRS* gene of *As. gossypii*. The numbers indicate the identity of the individual primary transformant. Asterisks indicate the lines that were further selected. C, control plants transformed with an empty vector.

Two different *PRS* genes of fungal origin were used for expression in plants. One encodes the wild-type PRS class I activity of *As. gossypii* ATCC 10895 (AGR371Cp) and the other represents a mutant form. The mutant variant carries three point mutations leading to the exchange of leucine 133 by isoleucine and histidine 196 by glutamine; the exchange of these two amino acids results in an alteration of the allosteric mechanisms regulating both enzyme inhibition by purine nucleotides and activation by inorganic phosphate (Becker *et al*., 1995). Therefore, this mutant form of the *As. gossypii* PRS protein resembles a protein of PRS class II activity. *As. gossypii PRS* genes were chosen because both variants of the gene were available that were highly heterologous to plant genes. All constructs used to transform either *N. tabacum* or *A. thaliana* plants were prepared with the binary vector pBinAR (Höfgen and Willmitzer, 1990), a derivative of the pBin19 vector containing the 35S promoter of the cauliflower mosaic virus for constitutive expression of the target gene and the octopine synthase polyadenylation signal. Primary transformants (T_1_) were grown on selection medium containing 50 mg/L kanamycin. Kanamycin-resistant plantlets were transferred to soil and grown in growth chambers under standard cultivation conditions. For each transformation, about 30 plants that had survived the selection process were regenerated and analysed further. The leaves of these plants were analysed for the expression of the transgene 3–5 weeks after the transfer (Figure 1). Seeds were collected, and the T_2_ offspring were grown on selection medium. Resistant plants of the T_2_ generation were selected when about one-quarter of the offspring were unable to survive the selection process. These plants were transferred to soil in growth chambers and further grown under optimum conditions, and the seeds were harvested (T_3_). The T_3_ seeds were again transferred to selection plates to identify the seed sets in which all plants were kanamycin resistant and could be regarded as homozygous for at least one functional insertion of the T-DNA. All experiments were carried out with seed from the T_3_ or T_4_ generation that had passed this selection process. Three to four lines originating from different individual primary transformants were chosen for each experiment. All experiments were carried out with control plants that had passed the same selection criteria after transformation with an empty vector.

### *PRS* expression leads to a significant but not severe increase in extractable PRS activity

It was confirmed that the expression of the *PRS* gene resulted in an increase in enzyme activity by assaying PRS activity using a standardized enzyme-coupled spectrophotometric determination procedure. This assay does not allow separate analysis of class I and class II PRS activities. As plant crude enzyme extracts contain both class I and class II PRS activities (Krath and Hove-Jensen, 1999), the plant enzyme activity cannot be distinguished from the additional *Ashbya* activities.

**Figure 2:**
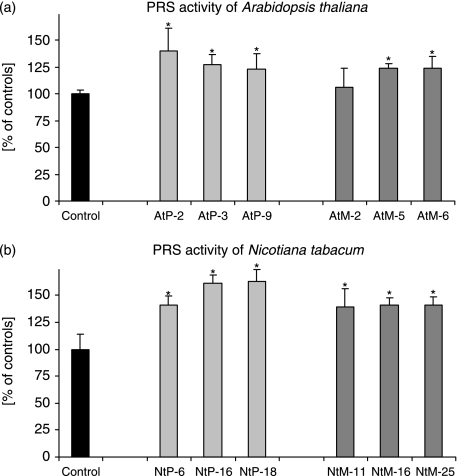
Phosphoribosylpyrophosphate synthetase (PRS) activities of *Arabidopsis thaliana* and *Nicotiana tabacum* seedlings. Plants were grown in liquid seedling culture for 7 or 8 days in their respective growth media. The experiments were carried out with plants of the T_4_ generation. Values are the means ± standard deviation of three to four lines originating from different individual primary transformants. Data are given as percentages of the respective control transformants, with *N. tabacum* at 2.17 ± 0.31 µmol/min/g fresh weight and *A. thaliana* at 2.83 ± 0.14 µmol/min/g fresh weight. Unpaired two-tailed *t*-tests were used. Significantly different values (*P* < 0.05) are labelled with an asterisk. (a) PRS activity of *Arabidopsis*. AtP, *A. thaliana* expressing the wild-type *PRS* gene; AtM, *A. thaliana* expressing a mutant form of the *PRS* gene; numbers indicate the identity of the individual primary transformant. (b) PRS activity of *Nicotiana*. NtP, *N. tabacum* expressing the wild-type *PRS* gene; NtM, *N. tabacum* expressing a mutant form of the *PRS* gene; numbers indicate the identity of the individual primary transformant.

The total PRS activity was assayed in soluble protein extracts of *A. thaliana* and *N. tabacum* seedlings grown for 7 or 8 days in liquid culture in their respective growth media (Figure 2). All data were calculated on a plant fresh weight basis as a percentage of the respective control transformants (*N. tabacum*, 2.17 ± 0.31 µmol/min/g fresh weight; *A. thaliana*, 2.83 ± 0.14 µmol/min/g fresh weight). Data were also calculated on the basis of soluble protein; as the soluble protein concentration on a fresh weight basis was unchanged between all analysed plants, the results are equivalent (data not shown). Our results showed that expression of the wild-type *PRS* gene significantly increased PRS activity (1.2–1.4-fold in *Arabidopsis* and 1.4–1.6-fold in *Nicotiana*). The increase was of the same magnitude in independent transformants, with no statistically significant differences between the individual lines. Expression of the mutated *PRS* gene increased the PRS activity by 1.3-fold in *Arabidopsis* and 1.4-fold in *Nicotiana*. With the exception of line AtM-2, the values showed no strong variation between the individual seed batches analysed, and were significantly higher when compared with those of the respective control transformants.

Our experiments were carried out on plants of the T_4_ generation, complying with the selection criteria described above for plants that could be regarded as homozygous for at least one functional insertion of the T-DNA. Below a certain number of identical transgenes in the genome, the gene copy number and expression are positively correlated (Schubert *et al*., 2004). Therefore, stable expression of the *PRS* gene over the analysed generations and a comparable level for the independent transformants are to be expected, and are reflected in the very low variation of increased PRS activity.

### Increased PRS activity increases fresh weight accumulation of seedlings in liquid culture

**Figure 3:**
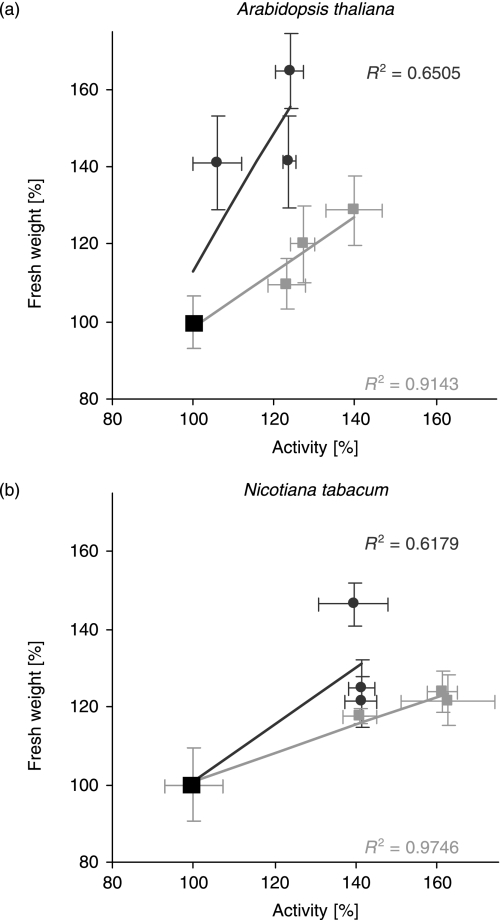
Correlation of phosphoribosylpyrophosphate synthetase (PRS) activity and fresh weight accumulation. Plants were grown in liquid seedling cultures for 7 or 8 days in their respective growth media as in Figure 2. Values are the means ± standard error of three to four lines originating from different individual primary transformants. Data are given as percentages of the respective control transformants, with *N. tabacum* at 49.6 ± 9.03 mg/seedling and *A. thaliana* at 2.7 ± 0.48 mg/seedling. Unpaired two-tailed *t*-tests were used. All fresh weight values are significantly different from the controls (*P* < 0.05), except for AtP-9. Linear correlation analysis was performed and the respective correlation coefficients are given. (a) Relationship of PRS activity and fresh weight accumulation of *Arabidopsis*: grey, *A. thaliana* expressing the wild-type *PRS* gene; black, *A. thaliana* expressing the mutant form; black square, empty vector controls. (b) Relationship of PRS activity and fresh weight accumulation of *Nicotiana*: grey, *N. tabacum* expressing the wild-type *PRS* gene; black, *N. tabacum* expressing the mutant form; black square, empty vector controls.

To investigate the influence of increased PRS activity on overall plant performance and growth, the fresh weight accumulation of seedlings in the T_4_ generation was measured in a highly controlled environment. Seedlings of *A. thaliana* and *N. tabacum* were grown in liquid cultures in their respective growth media, and plants were harvested and analysed after 7 or 8 days of growth. Expression of the native *PRS* gene and the mutant form in either *A. thaliana* or *N. tabacum* led to a significant increase in fresh weight accumulation in these young seedlings. Figure 3a shows that expression of the wild-type *PRS* gene, and the subsequent increase in PRS activity, in *Arabidopsis* significantly increased fresh weight accumulation by 1.1–1.3-fold. Expression of the wild-type *PRS* gene, and the subsequent increase in PRS activity, in *Nicotiana* had the same effect, resulting in significantly increased fresh weight accumulation of 1.17–1.25-fold (Figure 3b). A comparison of the PRS activity and fresh weight accumulation revealed a clear correlation of increased enzyme activity and increased fresh weight accumulation in both species (*R*^2^ > 0.9). Expression of the mutated *PRS* gene, which led to a less pronounced but nevertheless significant increase in PRS activity, resulted in an increased fresh weight accumulation of 1.4–1.6-fold in *Arabidopsis* and 1.2–1.5-fold in *Nicotiana*. Correlation of the PRS activity and fresh weight accumulation revealed an obvious relationship between increased enzyme activity and increased biomass (*R*^2^ > 0.6).

Some of the seedlings were harvested and the roots and shoots were analysed separately. No significant change in root to shoot ratio was found in any of the cultures and transformants for either *A. thaliana* or *N. tabacum* (data not shown), indicating that fresh weight accumulation increases in both organs of the different seedlings in parallel.

With our activity measurements, it was not possible to distinguish between the different endogenous and *As. gossypii* activities. Furthermore, with these measurements, it was not possible to differentiate between the diverse kinetic parameters of the enzymes. Nevertheless, these kinetic parameters must have an influence as, in both *Arabidopsis* and *Nicotiana*, the expression of the mutated form of *PRS* led to a less pronounced overall increase in PRS activity but a greater enhancement of biomass accumulation. Further studies of the different kinetic parameters and their influence on the metabolic flux through the pathway under various conditions are needed to explain this.

### Seed composition is unchanged in plants with ectopic expression of heterologous PRS activity in the cytosol

**Figure 4:**
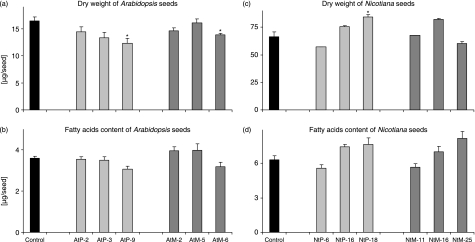
Seed composition analysis of *Arabidopsis thaliana* and *Nicotiana tabacum*. Prior to the harvest of fully developed seeds, plants of the T_3_ generation were grown on soil under long-day conditions as indicated in ‘Experimental procedures’. Values are given as means and standard errors of three biological replicates with seven (*Arabidopsis*) or four (*Nicotiana*) samples each. Unpaired two-tailed *t*-tests were used. Significantly different values (*P* < 0.05) are labelled with an asterisk. AtP, *A. thaliana* expressing the wild-type phosphoribosylpyrophosphate synthetase (*PRS*) gene; AtM, *A. thaliana* expressing a mutant form of the *PRS* gene; NtP, *N. tabacum* expressing the wild-type *PRS* gene; NtM, *N. tabacum* expressing a mutant form of the *PRS* gene; numbers indicate the identity of the individual primary transformant. (a) Seed dry weight of *Arabidopsis* seeds. (b) Fatty acid content of *Arabidopsis* seeds. (c) Seed dry weight of *Nicotiana* seeds. (d) Fatty acid content of *Nicotiana* seeds.

The higher fresh weight accumulation of the seedlings with additional PRS activity after growth in liquid culture may have two different explanations: (i) an increase in seed size as a result of larger amounts of storage compounds or an advantageous storage compound composition; (ii) a difference in metabolic activity in the growing seedling. To investigate whether increased PRS activity alters seed composition, *A. thaliana* and *N. tabacum* seeds were analysed for their individual seed weight and the content of their storage product fatty acids. The results from seeds from the same batches as used in the seedling culture experiments are shown. Expression of the *PRS* gene and mutant form in *A. thaliana* did not alter seed weight; furthermore, lines AtP-9 and AtM-6 showed a significant decrease (Figure 4a). The seed weight of PRS-expressing *N. tabacum* was highly variable between the individual lines, with a statistically significant increase in line NtP-18 (Figure 4c). These same results and high variance were also found in seeds of the T_3_ generation (data not shown). When all PRS-expressing lines were treated as biological replicates, no statistically significant difference was found between control lines and PRS expressers. The total fatty acid content of seeds of the T_4_ generation was unchanged in both *A. thaliana* and *N. tabacum* expressing the *PRS* gene. Within all analysed samples, there was no difference in fatty acid composition when compared with the respective control transformants (data not shown).

In all seeds analysed, the proportion of fatty acids of the individual seeds of *A. thaliana* was between 21% and 25%. These values are lower than those reported in other studies (O'Neill *et al*., 2003), and are presumably a consequence of the growth conditions. It has been shown previously that abiotic factors, mainly light intensity, have a strong influence on lipid accumulation of *A. thaliana* seeds (Li *et al*., 2006), with low light intensities causing lower seed weight and lipid accumulation. All of our *A. thaliana* plants for seed production and seed analysis were grown in a long-day phytotron at light intensities of only 145 µmol/m^2^/s, which might explain the low proportion of fatty acids in the seeds. In addition, the values for oil accumulation in all *N. tabacum* transformants were lower than those in other studies (Tomlinson *et al*., 2004). It is assumed that *N. tabacum* plants normally grown in high-light conditions are also limited in fatty acid accumulation when grown in a glasshouse with additional illumination of only approximately 200 µmol/m^2^/s.

As the increase in PRS activity has no significant influence on the weight of individual seeds or the proportions of storage compounds in seeds, the enhancement of seedling growth in liquid culture is unlikely to be the result of an enhanced supply of storage products.

### Metabolite analysis reveals a negative correlation of sucrose content with biomass accumulation

PRS is required for the synthesis of PRPP. This metabolite is a key intermediate, and is essential for nucleotide biosynthesis (Zrenner *et al*., 2006), the salvaging of nucleobases (Allen *et al*., 2002; Noctor *et al*., 2006) and tryptophan and histidine synthesis (see ‘Introduction’). Sufficient availability of PRPP is a crucial prerequisite for any metabolic process and, in particular, in dividing tissues, where it is an important metabolite for the production of nucleotides needed for DNA replication. To investigate whether increased fresh weight accumulation caused by increased PRS activity leads to changes in metabolic composition, extracts of the seedlings were analysed for carbohydrate, nucleotide and amino acid content and composition. Metabolite levels were calculated on a fresh weight basis to analyse differences in concentrations and on a total seedling basis to summarize productivity.

**Figure 5:**
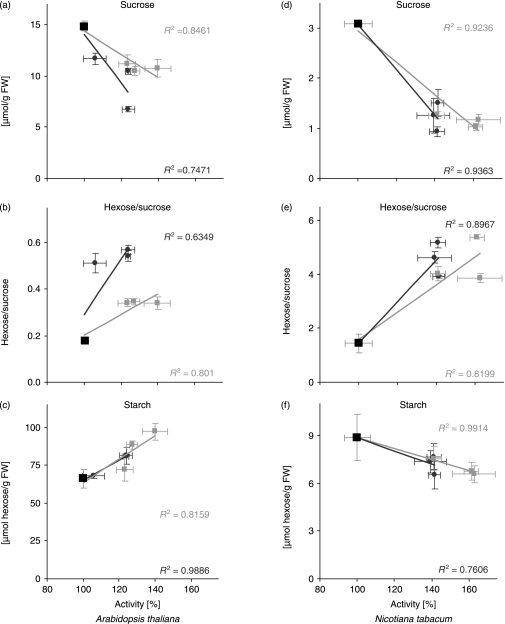
Carbohydrate analyses of *Arabidopsis thaliana* and *Nicotiana tabacum*. Carbohydrate contents of plants grown in liquid seedling culture for 7 or 8 days in their respective growth media, as described in Figure 2. Values are given as the means and standard errors of three to four replicates. Unpaired two-tailed *t*-tests were used. Linear correlation analysis was performed and the respective correlation coefficients are given. Hexoses: glucose and fructose. (a–c) Relationship of phosphoribosylpyrophosphate synthetase (PRS) activity and carbohydrate accumulation of *Arabidopsis*: grey, *A. thaliana* expressing the wild-type *PRS* gene; black, *A. thaliana* expressing the mutant form; black square, empty vector controls. All sucrose values (*P* < 0.05) and hexose/sucrose ratios (*P* < 0.01) are significantly different from the controls. (d–f) Relationship of PRS activity and carbohydrate accumulation of *Nicotiana*: grey, *N. tabacum* expressing the wild-type *PRS* gene; black, *N. tabacum* expressing the mutant form; black square, empty vector controls. All sucrose values (*P* < 0.05) and hexose/sucrose ratios (*P* < 0.05) are significantly different from the controls. FW, fresh weight.

Total carbohydrates (summarized as glucose, fructose, sucrose and starch on a fresh weight basis) were not changed significantly in either *A. thaliana* or *N. tabacum* seedlings with increased PRS activity (data not shown). Significant changes were found for the individual sugar levels, however. Although hexose concentrations were increased, the sucrose content declined significantly (Figure 5a,d), leading to a highly significant increase in the hexose-to-sucrose ratio in *A. thaliana* (Figure 5b) and *N. tabacum* (Figure 5e) seedlings. The decreased sucrose content and the increased hexose-to-sucrose ratio correlated well with the increased PRS activity in both *A. thaliana* and *N. tabacum* expressing either the wild-type or mutant *PRS* gene. The starch content was also correlated with the increased PRS activity, although these changes were not significantly different from the controls. In *A. thaliana*, the starch content increased with increasing PRS activity (Figure 5c); by contrast, the reverse was observed in *N. tabacum* (Figure 5f).

The carbon supply is highly co-ordinated with plant growth and, as a result of environmental changes, acute signalling and acclimatory responses can modulate plant growth (Smith and Stitt, 2007). However, the carbohydrate content of plants is highly variable, depending on fluctuations in the environment, such as day–night cycles, cold temperatures or drought treatment. In our seedling cultures, plants do not face environmental fluctuations as they were grown under continuous light with a fixed light intensity and temperature. Seedlings also grow independent of photosynthesis as they were supplied with adequate amounts of carbohydrates, reduced nitrogen and other essential macro- and micronutrients. Under these highly controlled and stable growth conditions, clear negative correlations between biomass and sucrose content were found, with correlation coefficients of *R*^2^ = 0.76 for *A. thaliana* and *R*^2^ = 0.60 for *N. tabacum*. This finding confirms the study of Meyer *et al*. (2007), who demonstrated that, under controlled growth conditions, the sucrose content (together with other metabolites) is negatively correlated with the biomass of *Arabidopsis* seedlings. It is also supported by the results of Cross *et al*. (2006), who showed that faster growing *Arabidopsis* accessions are characterized by lower levels of central metabolites such as carbohydrates. Although the starch content per milligram fresh weight of *A. thaliana* and *N. tabacum* seedlings did not change significantly, it correlated positively with increasing PRS activity in *A. thaliana* and negatively with increasing PRS activity in *N. tabacum*. Whether this opposite behaviour of starch accumulation reflects different strategies in the two species, or is just an expression of the less ideal growth conditions for *A. thaliana*, needs to be analysed in the future.

As the fresh weight accumulation of the seedlings with additional PRS activity was increased after growth in liquid culture, larger amounts of total carbohydrate content on a seedling basis were present in both *A. thaliana* and *N. tabacum* seedlings (data not shown). The increased total carbohydrate content of the seedlings is regarded as the result of a higher flux into major pathways of carbohydrate metabolism in seedlings with increased PRS activity.

**Figure 6:**
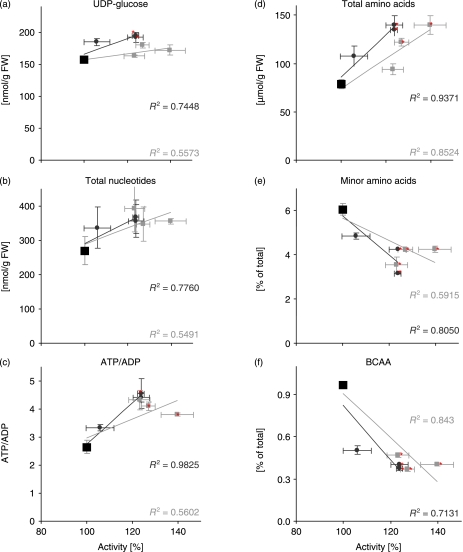
Metabolite analyses of *Arabidopsis thaliana* seedlings. Plants were grown in liquid seedling culture for 7 days, as described in Figure 2. Values are given as the means and standard errors of three to four replicates. Unpaired two-tailed *t*-tests were used. Significantly different values (*P* < 0.05) are labelled with a red asterisk. Linear correlation analysis was performed and the respective correlation coefficients are given. Total nucleotides: adenosine monophosphate (AMP), adenosine diphosphate (ADP), uridine diphosphate (UDP), guanosine diphosphate (GDP), uridine triphosphate (UTP), adenosine triphosphate (ATP) and guanosine triphosphate (GTP). Total amino acids: all l-α-amino acids without proline and cysteine, including β-alanine, γ-aminobutyric acid, citrulline and ornithine. Minor amino acids: arginine, histidine, isoleucine, leucine, lysine, methionine, phenylalanine, tryptophan, tyrosine and valine. BCAA, branched-chain amino acids: isoleucine, leucine and valine. (a–c) Relationship of phosphoribosylpyrophosphate synthetase (PRS) activity and nucleotide accumulation: grey, *A. thaliana* expressing the wild-type *PRS* gene; black, *A. thaliana* expressing the mutant form; black square, empty vector controls. (d–f) Relationship of PRS activity and amino acid accumulation: grey, *A. thaliana* expressing the wild-type *PRS* gene; black, *A. thaliana* expressing the mutant form; black square, empty vector controls. FW, fresh weight.

Other important precursors needed for cell division and growth are nucleotides and amino acids. Therefore, these intermediates were determined in *A. thaliana* and *N. tabacum* seedling cultures. Only the data from *A. thaliana* seedlings are shown (Figure 6), as significant changes correlating with increased PRS activity were only found in this species; *N. tabacum* seedlings showed comparable, but insignificant, tendencies.

The analysis of nucleotide concentrations revealed an increase in uridine diphosphoglucose (UDP-glucose) (Figure 6a) and free nucleotides (Figure 6b) in *A. thaliana* seedlings with increasing PRS activity, but only some of the UDP-glucose values were significantly different from the controls. In addition, in some of the *A. thaliana* seedlings, the ATP/ADP ratio was significantly increased (Figure 6c), but this ratio was decreased in *N. tabacum* seedlings with increasing PRS activity (data not shown). At this point, it is not known whether the different changes in the ATP/ADP ratios reflect a different energy state in the two species. Further analysis of nucleotide concentrations in relation to growth rates of different plant species is needed to elucidate possible correlations.

These results suggest that an increase in the overall nucleotide pool is not the main reason for the increased rate of growth in plants that over-express *PRS*. However, changes in flux through a pathway are not necessarily accompanied by corresponding changes in the levels of metabolic intermediates of the pathway (Fernie *et al*., 2005). Indeed, as already shown previously, nucleotide pools in fully grown organs are unaltered in transgenic plants with reduced pyrimidine *de novo* synthesis (Schröder *et al*., 2005). In these cases, the reduced availability of nucleotides is compensated by reduced growth rates, without alterations in nucleotide pools. In this context, it should also be noted that the measurements of metabolites were made over the entire seedlings, and may not reflect the levels in dividing and growing cells. Assuming that the increase in growth is somehow accomplished by higher division rates, higher fluxes through nucleotide biosynthetic pathways are expected to provide adequate amounts of nucleotides for DNA and RNA synthesis.

The analysis of total amino acid concentrations revealed an increase in *A. thaliana* seedlings that correlated well with increasing PRS activity (Figure 6d), but only some of the values were significantly different from the controls. A more detailed analysis of amino acid composition revealed identical behaviour in *A. thaliana* and *N. tabacum* seedlings, but again only some results from *A. thaliana* were significant. Although the total amount of amino acids was increased in all seedlings with increased PRS activity, the proportion of minor amino acids decreased (Figure 6e). This decreased proportion of minor amino acids may indicate an increased demand for protein biosynthesis (Fritz *et al*., 2006; Osuna *et al*., 2007). The proportion of histidine, an amino acid whose biosynthesis requires PRPP, was not changed in any of the transformation lines (data not shown). The other amino acid that requires PRPP for its synthesis is tryptophan. The proportion of tryptophan in the total amino acid pool was increased in both *A. thaliana* and *N. tabacum* seedlings with increased PRS activity (data not shown). However, tryptophan biosynthesis occurs in plastids (Radwanski and Last, 1995), and an increase in PRS activity in the cytosol is not necessarily expected to increase PRPP availability in plastids. Further studies of the subcellular distribution of metabolites are needed to clarify this. Other changes included an increase in the major amino acids serine and glycine (data not shown), and a correlating decrease in the proportion of the branched-chain amino acids (BCAA), in *A. thaliana* seedlings with increased PRS activity (Figure 6f). At this point, it is assumed that these changes in the proportion of specific classes of amino acids in relation to the total amino acid pool are a result of their co-ordinated regulation within individual amino acid biosynthesis pathways.

To investigate whether increased PRS activity leads to an increase in the immediate product of catalysis, several attempts were made to extract and measure PRPP in these seedling cultures. Using standardized protocols for metabolite extraction, such as trichloroacetic acid (TCA)–ether or perchloric acid, no reliable amounts of PRPP were measurable from seedling cultures (Dancer and apRees, 1989; data not shown). Therefore, a rather indirect approach was used to indicate the better supply of PRPP for metabolism and growth.

### Recycling of nucleobases is increased in *A. thaliana* seedlings with higher PRS activity

**Figure 7:**
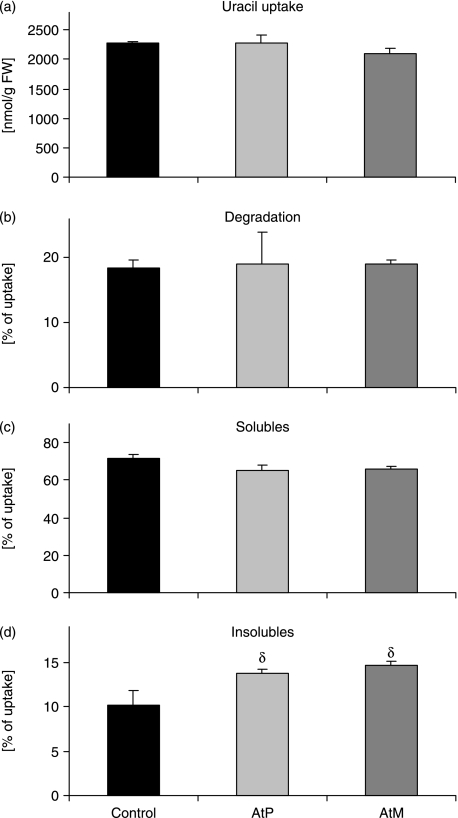
[2-^14^C]Uracil feeding to *Arabidopsis thaliana* seedlings. Plants were grown in liquid seedling culture for 7 days in their respective growth media, as described in Figure 2. [2-^14^C]Uracil was fed to the seedlings for 24 h and the plants were extracted as described in ‘Experimental procedures’. Values are given as the means and standard errors of three biological replicates. Unpaired two-tailed *t*-tests were used to compare data from the different transgenic material. Differences with *P* < 0.1 are labelled with δ. AtP, *A. thaliana* expressing the wild-type phosphoribosylpyrophosphate synthetase (*PRS*) gene; AtM, *A. thaliana* expressing the mutant form. FW, fresh weight.

To investigate whether increased PRS activity has an influence on nucleobase recycling in the cytosol, seedling cultures were incubated with labelled uracil and the metabolism of the isotope was monitored in various fractions (Figure 7). The higher PRS activity in *A. thaliana* transformants had no influence on the total uptake of uracil, or the amount and proportion of uracil that was immediately degraded to CO_2_. When the label of soluble metabolites, including unprocessed uracil and nucleotides, was separated from the label of the insoluble fraction containing DNA, a shift of the flux of the label into the insoluble fraction was observed in transformed seedlings with increased PRS activity. Although the difference in flux in the insoluble fraction was not highly significant (*P* < 0.1), a clear increase in the amount of radioactivity distributed in the insoluble fraction (from 10% of the total uptake for control transformants to 15% of the total uptake for the seedlings) with increased PRS activity was observed. It is concluded that the increased activity of PRS enhances the availability of PRPP in the cytosol for salvaging processes.

Nucleotide *de novo* synthesis pathways are mainly located in plastids (Zrenner *et al*., 2006). So far, no evidence has been found for a transport system exchanging PRPP between plastids and the cytosol. Therefore, an increase in PRS activity in the cytosol, and the subsequent production of PRPP in this compartment, is not necessarily expected to increase PRPP availability in plastids for nucleotide *de novo* synthesis. In the cytosol, several PRPP-consuming reactions are present. These include salvage pathways for nucleobases using the respective phosphoribosyltransferases (Allen *et al*., 2002; Islam *et al*., 2007) and the recycling of pyridine nucleotides (Noctor *et al*., 2006). In our uracil feeding experiment, a higher flux through the uracil phosphoribosyltransferase reaction in the cytosol is assumed to increase the recycling of the nucleobase uracil, finally leading to an increased incorporation of pyrimidine nucleotides into the insoluble fraction, namely into DNA or RNA.

### Higher biomass accumulation is also evident under different standardized growth conditions

**Figure 8:**
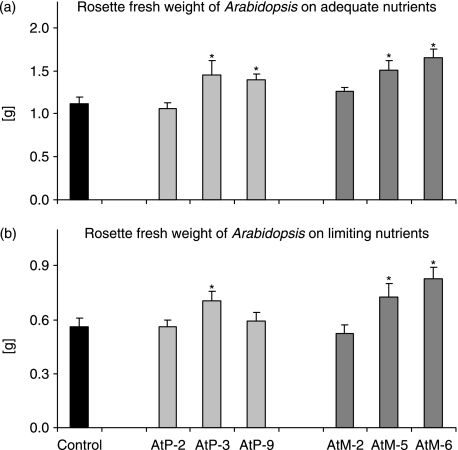
Growth analyses of *Arabidopsis thaliana* plants. Plants were grown in the different conditions as indicated. The experiments were carried out with plants of the T_4_ generation. Values are the means ± standard error of three biological replicates with 6–12 samples each. Unpaired two-tailed *t*-tests were used. Significantly different values (*P* < 0.05) are labelled with an asterisk. AtP, *A. thaliana* expressing the wild-type phosphoribosylpyrophosphate synthetase (*PRS*) gene; AtM, *A. thaliana* expressing a mutant form of the *PRS* gene; numbers indicate the identity of the individual primary transformant. (a) Rosette fresh weight of *Arabidopsis* plants grown with adequate nutrients. Plants were grown in growth chambers (8 h day at 145 µmol/m^2^/s, 20 °C, 60% relative humidity; 16 h night at 18 °C) in pots of 6 cm in diameter filled with a substrate containing high nutrient concentrations. Plants were harvested 5 weeks after transfer to soil. (b) Rosette fresh weight of *Arabidopsis* plants grown with limiting nutrients. Plants were grown with the same growth conditions and in parallel with plants in (a), but the substrate mixture contained low nutrient concentrations as described in ‘Experimental procedures’. Plants were harvested 5 weeks after transfer to soil.

**Figure 9:**
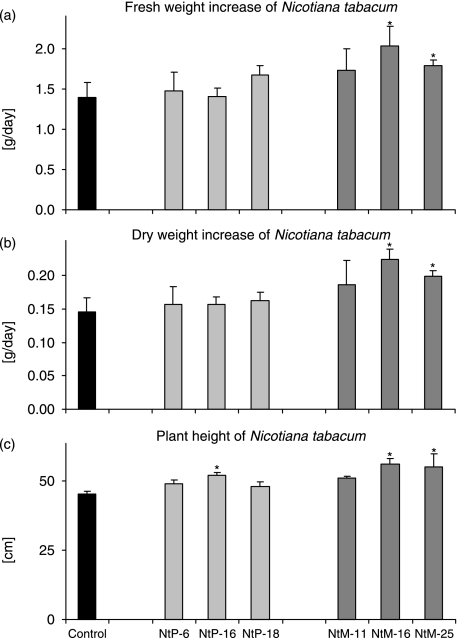
Growth analyses of *Nicotiana tabacum* plants. The experiments were carried out with plants of the T_4_ generation. Values are the means ± standard error of three biological replicates with four samples each. Unpaired two-tailed *t*-tests were used. Significantly different values (*P* < 0.05) are labelled with an asterisk. NtP, *N. tabacum* expressing the wild-type phosphoribosylpyrophosphate synthetase (*PRS*) gene; NtM, *N. tabacum* expressing a mutant form of the *PRS* gene; numbers indicate the identity of the individual primary transformant. (a) Fresh weight increase. Plants were grown in growth chambers (12 h day at 350 µmol/m^2^/s, 23 °C; 12 h night at 20 °C; 60% relative humidity) in pots of 16 cm in diameter in quartz sand culture, and watered daily with nutrient solution. Plants were harvested 3 and 4 weeks after transfer to sand culture, and the growth rates per day were calculated from these measurements. (b) Dry weight increase of *Nicotiana* plants grown as described in (a). (c) Height of *Nicotiana* plants. Plants were grown in a glasshouse (16 h day at 200 µmol/m^2^/s, 25 °C; 8 h night at 20 °C; 60% relative humidity) in pots of 20 cm in diameter filled with soil. Plants were measured 5 weeks after transfer to soil.

As shown previously, increased PRS activity increases the growth of *A. thaliana* and *N. tabacum* seedlings under optimized conditions in liquid culture (Figure 3). Further experiments were performed to investigate whether growth enhancement was also present in *A. thaliana* and *N. tabacum* plants grown under more natural and less optimized conditions. *A. thaliana* plants were grown in growth chambers on soil with two different nutrient regimes, and *N. tabacum* plants were grown either in growth chambers on quartz sand watered with nutrient solution at medium light intensities or in the glasshouse on soil with low light intensities. Expression of the *PRS* gene or mutant form in either *A. thaliana* (Figure 8) or *N. tabacum* (Figure 9) led to an increase in growth at all tested growth conditions. At both high and low nutrient availability, *A. thaliana* plants expressing the *PRS* gene showed a larger rosette diameter (data not shown) and higher rosette fresh weight when compared with the respective controls (Figure 8), whereas the leaf number was not altered (data not shown). *N. tabacum* plants expressing the *PRS* gene also showed increased biomass accumulation on a fresh and dry weight basis (Figure 9) and increased leaf area (data not shown). The fresh weight accumulation of roots and shoots was analysed separately, but no significant change in the root to shoot ratio was found (data not shown). Again, this indicates that growth increases in both organs in parallel. At all of the analysed growth conditions, randomized *N. tabacum* plants expressing the *PRS* gene could be easily distinguished from control transformants because of the increase in plant height. In summary, all of these approaches show that increased PRS activity increases plant biomass accumulation under a variety of growth conditions.

## Conclusion

Expression of *As. gossypii* genes coding for PRS, or a mutated variant of the same gene, increases the PRS activity of *A. thaliana* and *N. tabacum.* Increased PRS activity leads to a substantial increase in plant biomass accumulation under different growth conditions in both plant species. Our analysis provides evidence that the supply of PRPP co-limits the growth rates. It is postulated that increased PRS activity increases PRPP accessibility in the cytosol, which promotes nucleotide availability by enhancing nucleotide salvage processes. Further studies are needed to confirm these results under field conditions and to investigate the impact on the total seed yield.

The strategy of increasing the availability of precursors of basic metabolic processes, such as nucleotide biosynthesis, has obvious implications. It can be regarded as part of the biotechnological approach to increase plant biomass as an alternative renewable energy source.

## Experimental procedures

### Plant growth

The *A. thaliana* seedling culture was performed according to Scheible *et al*. (2004). *Arabidopsis* seeds (100–120) were surface sterilized and imbibed at 5 °C in complete darkness for 3 days. The seeds were transferred and grown in sterile liquid culture (250-mL Erlenmeyer glass flasks) on orbital shakers with constant, uniform fluorescent light (approximate photon flux density of 50 µmol/m^2^/s in the flask) and constant temperature (22 °C) in 30 mL of medium. The sterile full nutrition medium contained: 2 mm KNO_3_, 1 mm NH_4_NO_3_, 1 mm glutamine, 3 mm KH_2_PO_4_/K_2_HPO_4_ at pH 5.8, 4 mm CaCl_2_, 1 mm MgSO_4_, 2 mm K_2_SO_4_, 3 mm 2-(*N*-morpholino)ethanesulphonic acid (MES) at pH 5.8 (KOH), 0.5% (w/v) sucrose, 50 mg/L kanamycin, 40 µm Na_2_FeEDTA (EDTA, ethylenediaminetetraacetate), 60 µm H_3_BO_3_, 14 µm MnSO_4_, 1 µm ZnSO_4_, 0.6 µm CuSO_4_, 0.4 µm NiCl_2_, 0.3 µm HMoO_4_ and 20 nm CoCl_2_. The shaker speed was low (30 r.p.m.) during the first 3 days, and was then increased to 80 r.p.m. The seedlings were harvested after 7 days by quickly freezing in liquid nitrogen.

The *A. thaliana* plant culture on soil was performed as follows. Seeds were surface sterilized and aseptically grown on medium containing half-strength Murashige and Skoog salts (micro- and macro-elements including vitamins), 0.25 mm MES at pH 5.8 (KOH), 50 mg/L kanamycin, 0.5% (w/v) sucrose and 0.8% (w/v) agar. Seeds were imbibed at 5 °C in complete darkness for 3 days and grown in a 12-h photoperiod (photon flux density, 150 µmol/m^2^/s; 22 °C light; 18 °C dark). After 2 weeks, the plants were transferred to soil in pots of 6 cm in diameter. For the ‘adequate nutrients’ condition, the pots were filled with a 2 : 1 (v/v) mix of GS90 soil (composition: peat, clay, coconut fibre, 2 g/L salt, 160 mg/L N, 190 mg/L P_2_O_5_, 230 mg/L K_2_O, pH 6; supplied by Werner Tantau GmbH & Co. KG, Hetersen, Germany) and vermiculite (Gebrüder Patzer, Sinntal-Jossa, Germany), and grown under short-day conditions (8 h light, 16 h dark) at a light intensity of 145 µmol/m^2^/s, relative humidity of 60% and temperatures of 20 °C (day) and 18 °C (night). For the ‘limiting nutrients’ condition, the GS90 soil was replaced by a 1 : 10 (v/v) mix of GS90 soil and ‘Null-soil’ (composition: peat, clay, coconut fibre, 0.8 g/L salt, 50 mg/L N, 80 mg/L P_2_O_5_, 80 mg/L K_2_O, pH 6; supplied by Werner Tantau GmbH & Co. KG). Plants were grown under the same conditions as for the ‘adequate nutrients’ experiment.

For expression analysis and seed production and analysis, plants were grown in high-nitrogen conditions under a long-day (16 h light, 8 h dark) regime at a light intensity of 145 µmol/m^2^/s, relative humidity of 80% and temperatures of 20 °C (day) and 18 °C (night, 50% relative humidity).

The *N. tabacum* seedling culture was performed in an identical manner to the *Arabidopsis* seedling culture, but seedlings were harvested after 8 days and a different type of nutrient solution was applied. The tobacco full nutrition medium contained: Murashige and Skoog salts (micro- and macro-elements including vitamins), 0.25 mm MES at pH 5.8 (KOH), 50 mg/L kanamycin and 0.5% (w/v) sucrose.

The *N. tabacum* plant culture was performed as follows. Seeds were surface sterilized and aseptically grown on medium containing Murashige and Skoog salts (micro- and macro-elements including vitamins), 0.25 mm MES at pH 5.8 (KOH), 50 mg/L kanamycin, 0.5% (w/v) sucrose and 0.8% (w/v) agar. The seeds were imbibed at 5 °C in complete darkness for 3 days and grown in a 12-h photoperiod (photon flux density of 150 µmol/m^2^/s; 22 °C). After 4 weeks, the plants were transferred to pots of 20 cm in diameter filled with a 2 : 1 (v/v) mix of GS90 soil and sand in a glasshouse with a 16-h photoperiod (photon flux density of 200 µmol/m^2^/s; 25 °C light; 8 h night at 20 °C; 60% relative humidity). The plants were watered continuously using 100–250 mL of fertilizer-enriched water [Hakaphos spezial (16% N, 8% P, 22% K, 3% Mg) at a concentration of 1 g/L] (COMO GmbH & Co. KG, Münster, Germany) for each pot per day. Alternatively, after 4 weeks in tissue culture, plants were transferred to quartz sand (1 : 1 mix of particles with a size of 0.3–0.8 and 0.6–1.2 mm; Dorsilit Gebr. Schäfer, Mannheim, Germany) in pots of 16 cm in diameter in a climate chamber with a 12-h photoperiod (photon flux density of 350 µmol/m^2^/s; 23 °C light; 20 °C dark; 60% relative humidity). The pots were watered after approximately 3 h of illumination each day, filling the pot with nutrient solution and allowing it to run out, leaving the fluid retained between the sand grains (field capacity). The nutrient solution contained: 4 mm KNO_3_, 4 mm Mg(NO_3_)_2_, 3 mm KH_2_PO_4_/K_2_HPO_4_ at pH 5.8, 2 mm MgSO_4_, 1 mm NaCl, 40 µm Na_2_FeEDTA, 90 µm H_3_BO_3_, 20 µm MnSO_4_, 1.5 µm ZnSO_4_, 0.9 µm CuSO_4_, 0.6 µm NiCl_2_, 0.45 µm HMoO_4_ and 30 nm CoCl_2_.

### Cloning procedures and plasmid construction

*Escherichia coli* strain XL-1 Blue and *Agrobacterium tumefaciens* strain C58C1 containing pGV2260 were cultivated using standard procedures (Sambrook and Russell, 2001). Sequenz primers AgPRSv (GGATCCAATATGTCGTCCAAT) and AgPRSh (GGATCCTACATGACAGCG) were used to amplify the wild-type *PRS* from plasmid pJRAgprs1486 and the mutant *PRS* from pJRAgprs1404 (mutant) using standard procedures. Subcloning into pCR Script (Stratagene Europe Biocrest BV, Amsterdam Zuidoost, The Netherlands) was in accordance with the protocol provided by the supplier. Clones were confirmed by sequence analysis. The 965-bp *Bam* HI fragments encoding the full-length proteins were cloned into the *Bam* HI-restricted binary vector pBinAR (Höfgen and Willmitzer, 1990). The sense direction of the insertion was checked by *Sal* I digestion and polymerase chain reaction (PCR) analysis using the primers 35Shv (TATAGAGGAAGGGTCTTGCG) and AgPRSh. Definite plasmids to be transformed into plants were finally verified by sequence analysis.

### Plant transformation and expression analysis

*Agrobacterium*-mediated gene transfer was performed according to Rosahl *et al*. (1989) for tobacco plants and to Bent and Clough (1998) for *Arabidopsis*. Expression of the transgene was analysed by Northern hybridization according to Giermann *et al*. (2002), using the full-length wild-type *PRS* as a probe.

### Metabolite analysis

In liquid nitrogen, frozen plant material was ground to a powder using a ball mill (Retsch, Haan, Germany). Carbohydrates, amino acids and nucleotides were extracted and measured according to Schröder *et al*. (2005). Fatty acids were extracted according to the method of Bligh and Dyer (1959), and the lipid content was measured by gas chromatography of fatty acid methyl esters using pentadecanoic acid as internal standard (Benning and Somerville, 1992).

### Uracil feeding

The *A. thaliana* seedling culture was performed in full nutrition medium. After 7 days of growth, the seedlings were fed with uracil for 24 h under the same growth conditions by adding 1 µCi of [2-^14^C]uracil (7.4 GBq/mmol) at a final concentration of 2 mm to each flask. To measure the degradation of uracil, Whatman 3MM filter paper soaked with 3 m KOH was placed on top of each flask to capture [^14^C]-CO_2_. The seedlings were harvested in liquid nitrogen and frozen plant material was ground to a powder using a ball mill. The radioactivity of the filter papers was measured after 24 h of equilibration in water. Metabolites were extracted using an adapted perchloric acid extraction procedure (Katahira and Ashihara, 2002). Frozen plant material (250 mg) was immediately mixed with cold 10% perchloric acid containing 10 mm ethyleneglycol-bis(β-aminoethylether)-*N*,*N*′-tetraacetic acid (EGTA), and extracted for 30 min on ice. After centrifugation at 16 000 ***g*** for 5 min at 4 °C, the supernatant and pellet were separated. The pellet was washed with 250 µL of cold 80% ethanol and the supernatant, after centrifugation at 16 000 ***g*** for 5 min at 4 °C, was combined with the first supernatant and neutralized using 5 m KOH with 1 m triethanolamine. In this combined and neutralized supernatant fraction, radioactivity was measured for the soluble metabolites including nucleobases and nucleotides. In the pellet, the radioactivity of the insoluble fraction containing DNA and RNA was also determined.

### Enzyme activity

Frozen plant material was ground to a powder in liquid nitrogen using a ball mill. Aliquots of 10–20 mg fresh weight were extracted by vigorous vortexing with 500–1000 µL of extraction buffer. The composition of the extraction buffer was 50 mm KH_2_PO_4_/K_2_HPO_4_ at pH 7.5, 10% (v/v) glycerol, 0.1% (v/v) Triton X-100, 5 mm MgCl_2_, 1 mm ethylenediaminetetraacetic acid, 1 mm EGTA, 1 mm phenylmethylsulphonyl fluoride and 5 mm dithiothreitol. The extract was centrifuged at 16 000 ***g*** for 10 min at 4 °C. Measurements were performed in microplates by mixing 10 µL of the supernatant of the enzyme extract with 100–200 µL of measuring buffer (KH_2_PO_4_/K_2_HPO_4_ at pH 7.5, 5 mm MgCl_2_, 3.75 mm R5P, 2 mm ATP, 3.75 mm phosphoenolpyruvate, 0.2 mm NADH, 1.5 U myokinase, 3 U pyruvate kinase and 1.5 U lactate dehydrogenase). The soluble protein content of the supernatant was determined using a dye-binding assay (Bradford, 1976).
